# A Calcium- and GTP-Dependent Transglutaminase in *Leishmania infantum*

**DOI:** 10.3390/vetsci10030234

**Published:** 2023-03-20

**Authors:** Shawgi Hago Almugadam, Alessandro Trentini, Martina Maritati, Carlo Contini, Maria Cristina Manfrinato, Carlo Cervellati, Tiziana Bellini, Stefania Hanau

**Affiliations:** 1 Department of Neuroscience and Rehabilitation, University of Ferrara, Via Luigi Borsari 46, 44121 Ferrara, Italy; 2 Faculty of Medical Laboratory Sciences, University of Khartoum, Nile Avenue, P.O. Box 321, Khartoum 51111, Sudan; 3 Department of Environmental and Prevention Sciences, University of Ferrara, Via Luigi Borsari 46, 44121 Ferrara, Italy; 4 Infectious Diseases and Dermatology, Department of Medical Sciences, University of Ferrara, Via Aldo Moro 8, 44124 Ferrara, Italy; 5 Department of Translational Medicine and for Romagna, University of Ferrara, Via Luigi Borsari 46, 44121 Ferrara, Italy

**Keywords:** *Leishmania*, transglutaminase, protein cross-linking, calcium-dependent activity, GTP-dependent activity, transamidation

## Abstract

**Simple Summary:**

Protozoan parasites of the *Leishmania* genus (Kinetoplastida: Trypanosomatidae) are responsible for human and animal leishmaniasis, pathologies mainly spread in the tropical and subtropical regions of the Americas and Afro-Eurasia, with several millions of people affected worldwide. Antileishmanial drugs present problems associated with drug toxicity and increasing parasite resistance. Therefore, the acquirement of further knowledge of these parasites with a focus on new potential drug targets is extremely useful. While transglutaminases are known to be involved in cell death and autophagy, it appears that these functions are very important for parasites’ virulence. Transglutaminase activity has been reported in *Leishmania,* where it was shown to be important for the proliferation of the insect promastigote stage. However, the enzyme has never been purified or well characterized. This study aimed to fill this knowledge gap by isolating and characterizing TGase from *L. infantum* promastigotes. For the first time, we showed a Ca^2+^- and GTP-dependent TGase in *Leishmania* corresponding to a 54 kDa protein, which was purified to homogeneity through two chromatographic steps: DEAE-Sepharose and Heparin-Sepharose. These results might allow for the exploration of the potential of this enzyme in fighting leishmaniasis.

**Abstract:**

While human and animal leishmaniasis affect several millions of people worldwide, *L. infantum* is the species responsible for visceral leishmaniasis in Europe, Middle East, and America. Antileishmanial drugs present issues associated with drug toxicity and increasing parasite resistance. Therefore, the study of this parasite with a focus on new potential drug targets is extremely useful. Accordingly, we purified and characterized a transglutaminase (TGase) from *L. infantum* promastigotes. While Tgases are known to be involved in cell death and autophagy, it appears that these functions are very important for parasites’ virulence. For the first time, we showed a Ca^2+^- and GTP-dependent TGase in *Leishmania* corresponding to a 54 kDa protein, which was purified by two chromatographic steps: DEAE-Sepharose and Heparin-Sepharose. Using polyclonal antibodies against a 50-amino-acid conserved region of the catalytic core of human TGase 2, we revealed two other bands of 66 and 75 kDa. The 54 kDa band appears to be different from the previously reported TGase, which was shown to be Ca^2+^- independent. Future research should address the identification of the purified enzyme sequence and, subsequently, its cloning to more comprehensively investigate its pathophysiological function and possible differences from mammal enzymes.

## 1. Introduction

Protozoan parasites of the *Leishmania* genus (Kinetoplastida: Trypanosomatidae) are responsible for human and animal leishmaniasis, pathologies that are found to be endemic in Asia, the Middle East, North Africa, East Africa, the Mediterranean, and South and Central America [1,2]. Leishmaniasis affects more than 12 million people worldwide [2], with almost 250,000 new cases of both cutaneous and visceral leishmaniosis reported in 2021 (data source: World Health Organization, accessed on 8 March 2023, last updated on 6 February 2023; https://apps.who.int/neglected_diseases/ntddata/leishmaniasis/leishmaniasis.html).

While more than 70 animal species can be the source of this parasite, the well-recognized *Leishmania* hosts are humans and dogs, representing important reservoirs of *L. infantum*, the species responsible for visceral leishmaniasis in Europe, Middle East, and the Americas. The human infection can manifest as visceral, cutaneous, and/or mucocutaneous syndromes depending on many factors, including the involved species of *Leishmania* and the nutritional status of the host. One hundred species of phlebotomine sandflies can be vectors of *Leishmania* parasites, wherein the extracellular promastigote stage is carried in the gut. When the promastigote has been injected into a vertebrate, it mainly targets phagocytic cells, where it will transform into the amastigote replicating stage within the phagolysosomal compartment [3].

As an antileishmanial drug, allopurinol is mainly used for canine infection alone or in combination with antimonials or miltefosine [4]. Human leishmaniasis is treated with amphotericin B lipid-associated formulations, antimonials, pentamidine, paromomycin, miltefosine, and a few other compounds, such as ketoconazole for cutaneous forms. Only miltefosine has oral administration, and at variance,, all effective drugs present problems associated with drug toxicity and increasing parasite resistance. Therefore, the study of this parasite with a focus on new potential drug targets is extremely useful [5].

Transglutaminases (TGase, E.C. 2.3.2.13) are a family of enzymes catalyzing several post-translational modifications to proteins, including transamidation, esterification, and hydrolysis. The best-known reaction is transamidation, consisting of the formation of a lysine–glutamine isopeptide bond, leading either to the attachment of a polyamine to glutamine or protease-resistant protein cross-linking [6,7]. In addition, members of the TGase family can act as Ser/Thr kinases, GTP/ATP hydrolases, or protein disulfide isomerases (PDI) [8,9,10]. TGases have different subcellular localizations and are involved in a plethora of biological processes, including growth, differentiation, cell death, autophagy, cellular adhesion, and receptor-mediated endocytosis. In addition, these enzymes control signal transduction and promote blood/hemolymph coagulation and skin/cuticle formation [8,11]. For their transamidation activity, most of the TGases require, other than the catalytic cysteine, a calcium ion. On the contrary, nucleotides such as GTP, GDP, and ATP can inhibit TGase 2, 3, and 5 [12]. For instance, it has been shown that the binding of GTP or GDP to TGase 2 inhibits the enzyme, causing the stabilization of a compact conformation that blocks the access of substrates to the active site [13,14]. On the other hand, the binding of calcium to the enzyme induces a large conformational change, resulting in the formation of an extended structure with the active site exposed to substrates accompanied by a reduced affinity for GTP [13,14,15]. However, not all TGases are calcium-requiring-GTP-modulated enzymes, such as some microbial TGases [16,17,18]. For instance, consider the excreted microbial MTGase from *Streptomyces mobaraensis*, which has no sequence homology with mammalian TGases and a single domain structure, compared to the four-domain human TGase 2. Nonetheless, both the human and microbial TGases possess a Cys–His–Asp catalytic triad [18].

Previous reports showed the presence of a Ca^2+^-independent TGase activity in *Leishmania*. This isoenzyme was shown to play an important role in the proliferation of the promastigote stage [19]. Of note, one of the substrates of *Leishmania* TGase was GP63, also known as leishmanolysin or *Leishmania* Major Surface Protease, a zinc-dependent metalloprotease crucial for *Leishmania’s* survival and virulence in the host [20,21]. This finding provided further support for the critical role of TGase in the parasite’s life cycle.

Nevertheless, a complete characterization of the enzyme from a biochemical perspective is still unavailable in the literature. In particular, the enzyme has never been purified, its molecular mass (M) is still unknown, and no TGase-like sequences can be found in the genome of the parasite, implying that, as in other lower organisms, it might diverge from mammalian TGases [10,22]. Therefore, in this study, we sought to characterize and purify TGase from *L. infantum* promastigotes. Our findings suggest the presence of a Ca^2+^-requiring and GTP-modulated TGase. Furthermore, the purification of the enzyme to homogeneity via two-step chromatography allowed us to identify a major band at ≈54 kDa, which was determined by denaturing and reducing SDS-PAGE, whereas Western blot analysis using polyclonal antibodies against a 50-amino-acid conserved region of human TGase 2 revealed two other bands of a higher apparent mass, namely, 66 and 75 kDa.

## 2. Materials and Methods

### 2.1. Chemicals and Reagents

Fluorescein-cadaverine (FC, Molecular Probes^TM^ A10466) was purchased from Thermo Fisher Scientific (Waltham, MA, USA). Putrescin, 4’,6-diamidino-2-phenylindole (DAPI), TGase assay kit (CS1070-1KT), RPMI 1640 medium, and goat serum were from Sigma-Aldrich (Merck KGaA, Darmstadt, Germany). Diethyl aminoethyl (DEAE) and Heparin Sepharose were purchased from Cytiva (Marlborough, MA, USA) and Sigma-Aldrich, respectively, and nitrocellulose membrane (Amersham Protran Supported) was purchased from GE Healthcare (Chicago, IL, USA). Human TGase 2 rabbit polyclonal antibodies (orb2986) were purchased from Biorbyt (Cambridge, UK). Horseradish-peroxidase (HRP)-conjugated goat anti-rabbit secondary antibodies were purchased from Novus Biologicals Europe, UK. FITC-tagged anti-rabbit antibodies were obtained from Chemicon (Temecula, CA, USA). Fetal bovine serum (FBS, South America origin, EU Approved) was obtained from EuroClone, Italy. Protease inhibitor cocktail and the Western Blot detection system SuperSignal™ West Femto Maximum Sensitivity Substrate were obtained from Thermo Scientific (Waltham, MA, USA). Protein molecular mass markers ECL Plex Fluorescent Rainbow and Precision Plus Protein™ Dual Colour Standards were obtained from BIO-RAD (Hercules, CA, USA). All other chemicals were obtained from Sigma-Aldrich.

### 2.2. Parasites

The promastigotes of IZSLER_MO1 and MHOM/TN80/IPT1 *L. infantum* strains, which were kindly provided by IZSLER in Modena, Italy, were routinely cultured in RPMI 1640 medium at pH 7.2, which was supplemented with 15% heat-inactivated, 0.2 of micron-filtered FBS, 2 mM of L-glutamine, 100 U/mL of penicillin, and 100 U/mL streptomycin sulfate, in 25 cm^2^ non-vented flasks at 25 °C in a humidified incubator under an air atmosphere in the gas phase, for which the medium was replaced every 3–4 days [23].

### 2.3. Preparation of Lysates

Promastigotes (4 × 10^9^) were harvested by centrifugation at 1200× *g* at 4 °C, washed four times with phosphate-buffered saline (PBS), and the pellets were frozen at −80 °C. Cell lysis was performed with five volumes: weight of buffer containing 50 mM Tris/HCl at pH 7.4, 150 mM NaCl, 1% Triton X-100, 5 mM ethylenediaminetetraacetic acid (EDTA), 1 mM dithiothreitol (DTT), and protease inhibitor cocktail for 30 min on ice, followed by three freeze/thaw cycles and sonication in ice using Ultrasonic Processor XL Sonicator (Farmingdale, NY, USA) (five pulse cycles of 20 s at an intensity of 50% with five cooling pauses of 20 s). The cell homogenate was centrifuged at 13,000× *g* and 4 °C for 30 min to remove particulate, and the supernatant was dialyzed in cellulose membrane with a cutoff of 14 kDa against a buffer containing 50 mM Tris/HCl at pH 7.4, 150 mM NaCl, 5 mM EDTA, 1 mM DTT, and protease inhibitor cocktail and/or directly stored in aliquots at −80 °C. Total protein content was determined using the Bradford method.

### 2.4. In Vivo Detection of TGase Activity

Fluorescein-cadaverine (FC) was used according to a modified version of the method reported by Lajemi [24]. About 10^7^ of cultured *L. infantum* promastigotes were incubated in the dark for 3 h at 25 °C with 0.5 mM FC in complete RPMI medium. The parasites were then washed three times in PBS and then smeared on glass slides, air-dried, and fixed for 10 min at −20 °C with cold methanol to remove free intracellular FC [25]. The smears were then incubated for 20 min in the dark with 300 nM DAPI, washed for 10 min three times in PBS, mounted in glycerol in PBS (3:1, *v*/*v*) containing 0.1% 1,4-phenylenediamine, and examined using a Nikon Microphot FXA fluorescent microscope equipped with a camera. 

### 2.5. In Vitro in-Gel Detection of TGase Activity

Cell lysates were incubated for 1 h at 30 °C with 4 mM FC with or without 50 µL of 20 mg/mL dimethyl casein (prepared according to the method of Lin et al. (1969) [26]) and in the absence or presence of 200 µM putrescine under end-to-end roll mixing. The reaction products were then mixed with Loading Buffer (2% *w*/*v* SDS, 5% *v*/*v* β-mercaptoethanol, 10% *v*/*v* glycerol, 0.01% *w*/*v* bromophenol blue dye, and 0.5 M Tris-HCl at pH 6.8), boiled at 95 °C for 5 min, and then subjected to 15% polyacrylamide SDS-PAGE. Then, gel images were acquired with the Molecular Imager System PHAROS Bio-Rad FX and elaborated with Quantity One 1-D software (version 4.6.9).

### 2.6. In Vitro Micro-Well Plate Detection of TGase Activity

TGase activity was determined by the activity assay kit purchased from Sigma-Aldrich (Cat. No. CS1070-1KT) according to the manufacturer’s instructions. The binding of biotin-TVQQEL-OH peptide (biotynilated glutamine-containing peptide) in the assay buffer to the free amine group of poly-L-lysine covalently attached to the micro-well plate’s surface was measured via streptavidin–peroxidase reaction with 3,3′,5,5′-Tetramethylbenzidine (TMB) used as substrate and guinea pig liver TGase used as a positive control. Extracts, dialyzed extracts, protein fractions obtained after ammonium sulfate precipitation, and positive and negative controls were assayed in the absence or presence of ethylene glycol-bis (β-aminoethyl ether)-N,N,N′,N′-tetraacetic acid (EGTA), CaCl_2_, and GTP. Briefly, the substrate-coated plate was equilibrated to room temperature (RT); then, 50 µL of sample was loaded in each well and 50 µL of the assay mixture was added, gently mixed, and incubated at RT for 30 min. The assay mixture, which contained, for each reaction, 10 µL of the Assay Buffer, 1 µL of 1 M DTT, and 40 µL of ultrapure water, was prepared immediately before use. After incubation, 3 rinses were performed with ultrapure water; then, 100 µL of fresh 0.1 µg streptavidin-peroxidase in PBS at pH 7.4 with 0.05% TWEEN 20 and 1 mM DTT (PBS-T) was added to each well, and the plate was incubated at RT for 20 min. The wells were then washed three times (200 µL/well each) in PBS-T. Subsequently, 200 µL of TMB was added to each well and 100 µL of stop solution was added after a maximum of 3 min. Absorbance was read at 450 nm using Tecan Infinite M200 (Tecan Trading AG, Mannedorf, Switzerland). Experiments were repeated three times and each assay was conducted in triplicate. 

### 2.7. Immunocytochemical Staining

Indirect immunofluorescence microscopy was performed using the method of Upchurch with some modifications [27]. Briefly, 10^7^ promastigotes were washed twice in PBS. Parasite smears were prepared on clean, glass slides; air-dried; and fixed for 20 min at RT with 4% *w*/*v* paraformaldehyde in PBS. After being washed three times for 5 min in PBS, the slides were incubated for 30 min with 1% BSA in PBS to block non-specific binding sites on the glass. Smears were then incubated overnight at 4 °C with human TGase 2 rabbit polyclonal antibodies diluted 1:1500 in PBS containing 0.3% Triton X-100 and 0.1% goat serum. At the end of the incubation period, the smears were washed (a) three times for 5 min at RT with PBS; (b) three times for 10 min with 0.1% BSA in PBS; (c) one time for 30 min with 0.1% goat serum in PBS; and (d) 10 min in PBS. After all the washing steps were performed, the slides were incubated for 1 h in the dark with FITC-labeled anti-rabbit antibodies diluted 1:100 in PBS containing 0.3%Triton X-100 and 0.1% goat serum. After the incubation, the smears were washed three times for 10 min with 0.2% Triton X-100 in PBS and then incubated in the dark for 20 min with 300 nM DAPI. After a single washing procedure conducted for 10 min with 0.2% Triton X-100 in PBS, the smears were mounted in glycerol diluted with PBS (3:1, *v*/*v*) containing 0.1% 1,4-phenylenediamine and examined using a Nikon Microphot FXA fluorescent microscope equipped with a camera. 

### 2.8. TGase Purification

TGase purification was performed using a modified method based on the literature [28]. *Leishmania* extracts were first subjected to anion exchange chromatography on DEAE-Sepharose, equilibrated in 25 mM Tris buffer at pH 7.0, 150 mM NaCl, 2 mM EDTA, and 2 mM DTT (buffer A), and proteins were eluted by using buffer A plus additional 0.2 M NaCl. The eluted fractions were dialyzed against 25 mM Tris buffer at pH 7.0, 2 mM EDTA, and 2 mM DTT (buffer B) and subjected to affinity chromatography on Heparin-Sepharose equilibrated with buffer B. Elution was accomplished with buffer B plus 0.5 M NaCl.

### 2.9. Immunoblotting of Promastigote Lysates

For the detection of TGase in promastigote lysates, about 14 µg of proteins was separated by 10% polyacrylamide SDS-PAGE under reducing conditions and then transferred onto 0.45 µm pore nitrocellulose membranes using a wet system (Bio-Rad) at 300 mA and 150 V for 90 min using Tris-glycine-SDS blotting buffer with 20% methanol. Non-specific sites of the blots were blocked through incubation with 5% skim milk and 0.5% Tween-20 in PBS (blocking buffer) for 1 hr, and the blots were incubated overnight at 4 °C with rabbit polyclonal antibodies (1:1500 in 1% BSA, 0.5% Tween-20 in PBS) against a conserved region of TGase 2 enclosing a sequence in the catalytic domain of the enzyme. After executing three washing steps with PBS-T (0.1% Tween-20 in PBS), the blots were incubated with anti-rabbit-HRP secondary antibodies (1:50,000 in blocking buffer) for 1 h at RT and washed 3 times for 10 min each with PBS-T and 3 times for 10 min each with PBS. Finally, the bands were detected with the ECL Plex SuperSignal™ West Femto (Thermo Scientific).

### 2.10. Statistical Analyses

Statistical analyses were performed via one-way ANOVA with Bonferroni correction for multiple comparisons. All the analyses were carried out with GraphPad^®^ Prism 6 and a *p* < 0.05 was considered significant. 

## 3. Results

### 3.1. Detection of TGase Activity and Effect of Ca^2+^ and GTP 

**Figure 1:**
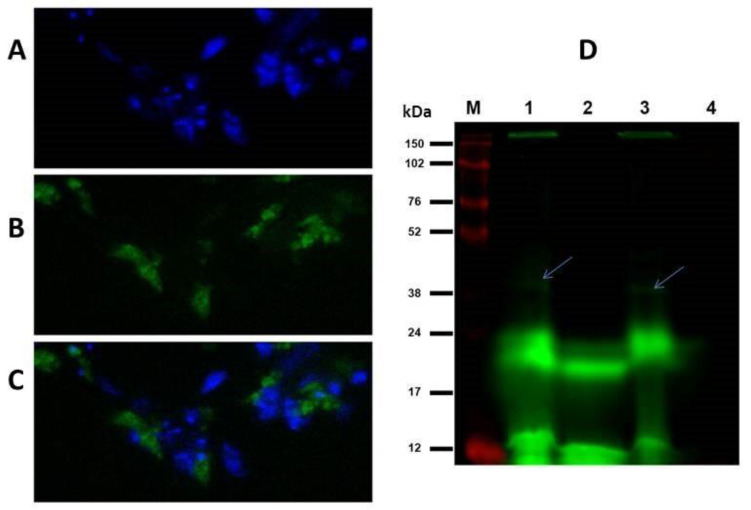
Detection of TGase activity in cultured promastigotes of *L. infantum* in vivo (**A**–**C**) and in vitro (**D**). (**A**) DAPI-stained nuclei of the parasites. (**B**) Fluorescein-cadaverine (FC) labelling of indigenous proteins via TGase activity. (**C**) Merged photo of A and B. Fluorescence was detected by Nikon Microphot FXA fluorescent microscope (×100 magnification). (**D**) 15% SDS-PAGE of *L. infantum* promastigotes lysate, labelled with FC. The green bands show excitation of the FC, and the red bands denote excited Cy5, which labels the molecular-mass marker. Molecular Imager System PHAROS Bio-Rad FX and software Quantity One 1-D were used for gel analysis. From left to right: lane M—molecular-mass marker; lane 1—parasite extract + FC + dimethyl casein (arrows); lane 2—parasite extract + FC; lane 3—parasite extract + FC + dimethyl casein+ putrescine; and lane 4—dimethyl casein alone (no fluorescence).

TGase-transamidating activity was confirmed both in vivo and in vitro in L. infantum promastigotes, which was revealed through the specific incorporation of fluorescein-cadaverine after the incubation of the cells with diamine for 3 h at 25 °C (Figure 1A–C) and of the parasite lysate for 1 h at 30 °C (Figure 1D). In the latter case, fluorescent protein bands under 24 kDa were predominant in the gel, suggesting the presence of several endogenous substrates. Nonetheless, when dimethyl casein was added to the mixture as an exogenous substrate, a very faint band around 38 kDa appeared (Figure 1D, lanes 1 and 3, wherein bands are indicated by arrows). The presence of TGase was further confirmed by the in vitro micro-well plate detection activity assay, demonstrating a quite low specific activity in the promastigotes of about 0.1 mU/mg, which was weakly inhibited by 200 µM of putrescine (data not shown).

**Figure 2:**
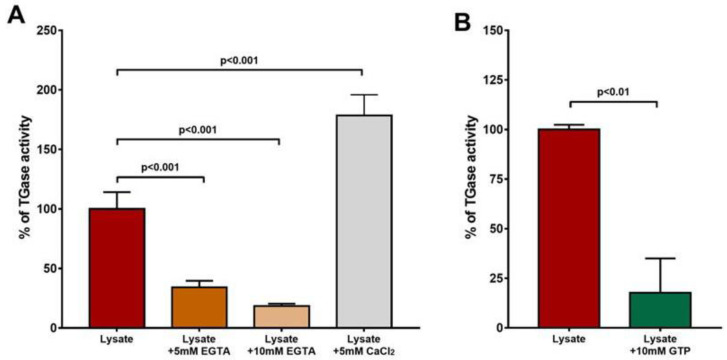
Ca^2+^ activation and GTP inhibition of *L. infantum* promastigotes’ activity. The percentages of the activities are represented. (**A**) Addition of EGTA to the reaction mix significantly reduced the enzyme’s activity (*p* < 0.001), and treatment of lysate with additional Ca^2+^ increased the enzyme’s activity. (**B**) Addition of GTP markedly reduced TGase activity.

An activity assay was used to better characterize the enzyme modulation by calcium and GTP. As displayed in Figure 2A, the addition of 5 and 10 mM of EGTA to the cell lysate caused a decrease in the level of detected activity, which was restored upon the supplementation of 5 mM of Ca^2+^. Moreover, by adding 10 mM of GTP to the lysate, we observed a decrease in enzymatic activity (Figure 2B). Together, these results suggest that the regulation of the activity of this enzyme is Ca^2+^- and GTP-dependent.

### 3.2. TGase Purification

**Figure 3:**
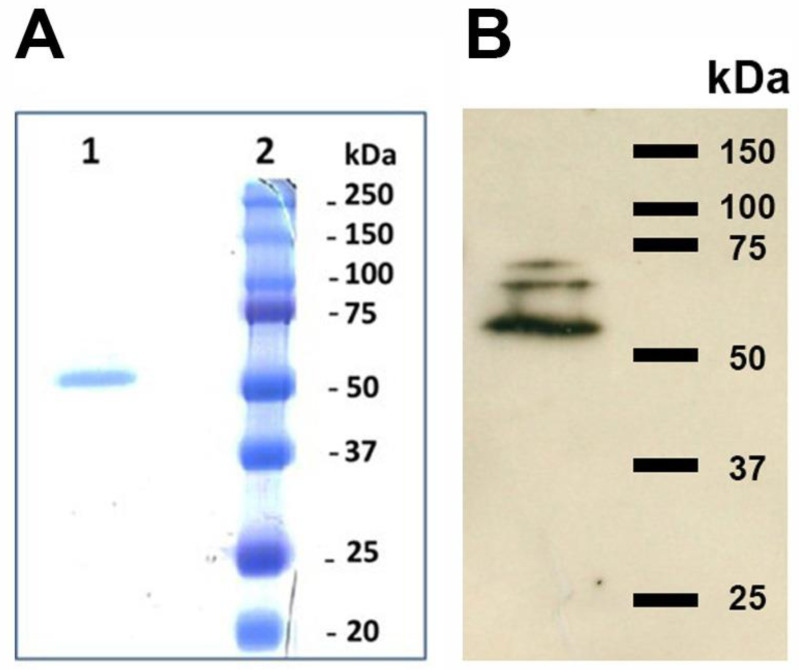
10% SDS-PAGE (panel **A**) of the purified TGase and Western blot (panel **B**). Lane 1, fraction obtained from the affinity chromatography on Heparin-Sepharose; lane 2, molecular mass marker. (please find the WB full membrane in Figure S1).

Several methods of TGase purification are known; these usually involve at least one step consisting of ion exchange chromatography on Diethylaminoethyl (DEAE)-Sepharose or carboxymethyl (CM)-cellulose followed by further purification via size exclusion chromatography and heparin-agarose affinity chromatography [29]. In particular, the latter step exploits the affinity of TGases for the medium, which, ideally, resembles the physiological binding of the enzyme to proteoglycans that has been shown in some experiments [28]. Therefore, we opted for a two-step chromatographic separation involving an initial purification on DEAE-Sepharose followed by affinity chromatography on heparin-agarose. After only two chromatographic separations, we obtained a band with an apparent mass of 54 kDa (Figure 3). The yield was 12%, affording 970 mU out of 8000 in the parasite extract and 4212 in the dialyzed eluted fractions from DEAE-Sepharose, while the final specific activity of 1687 mU/mg in the fractions eluted from Heparin-Sepharose was only 10-fold greater than those obtained with anionic exchange, suggesting possible inactivation during purification. In addition, the variance in the specific activity of the different extracts was very high.

### 3.3. Cross-Reactivity of L. infantum Promastigotes with Antibodies against Human TGase 2

**Figure 4:**
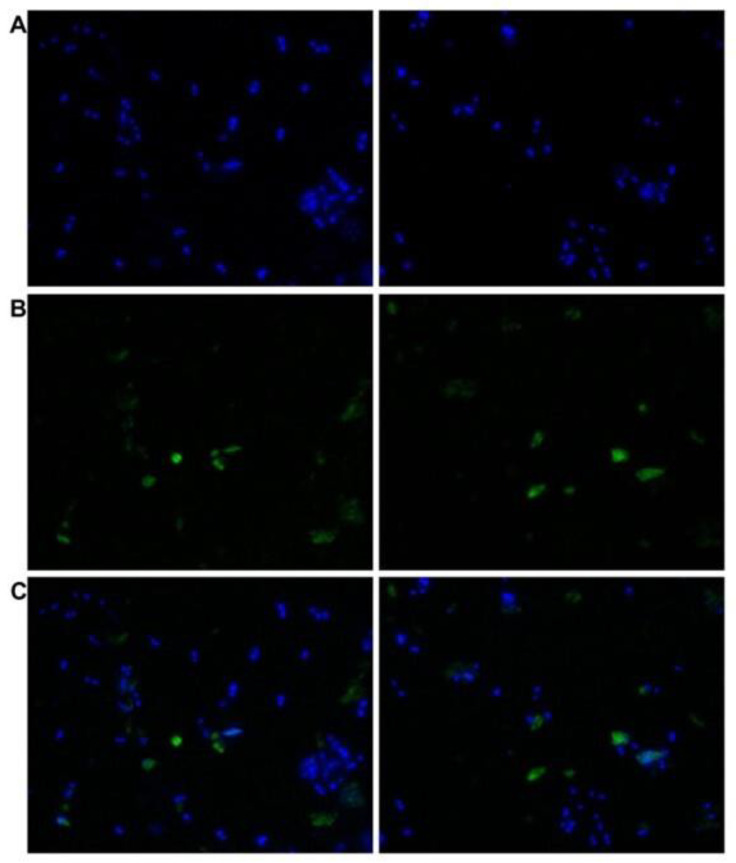
Indirect immunofluorescent staining of *L. infantum* promastigotes smeared on glass slides with human TGase 2 rabbit polyclonal antibodies (orb2986) and FITC-tagged anti-rabbit antibodies. FITC fluorescence was visualized using a Nikon Microphot FXA fluorescent microscope (×40 magnification). (**A**) DAPI-stained nuclei of the parasites. (**B**) FITC labelling. (**C**) Merged image of A and B.

**Figure 5:**
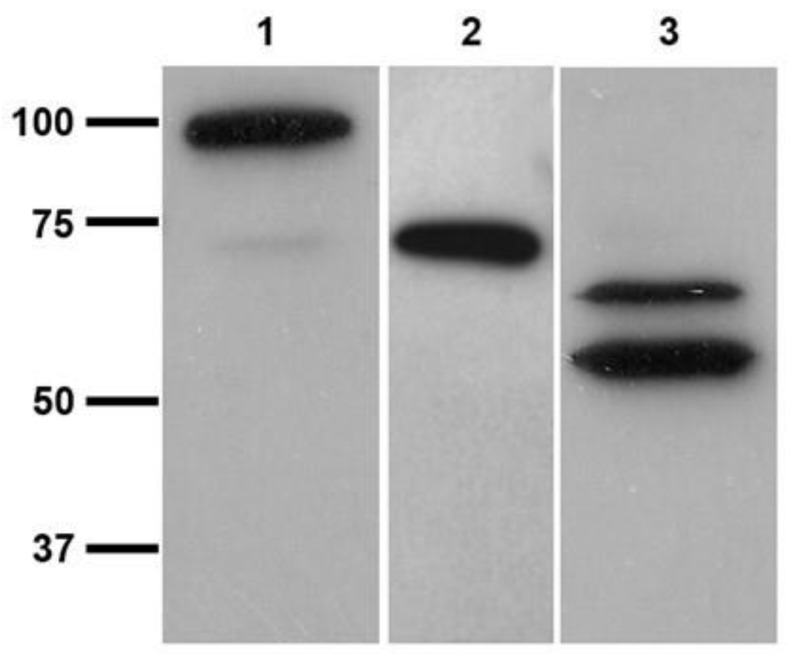
10% SDS-PAGE electrophoresis and immunoblotting of *L. infantum* promastigotes lysates using transglutaminase polyclonal antibodies (orb2986), HRP-conjugated Goat anti-Rabbit (H + L) secondary antibodies, and ECL detection system. Lane 1, purified guinea pig TGase; lane 2, IZSLER_MO1 *L. infantum* extract; and lane 3, MHOM/TN80/IPT1 *L. infantum* extract. (please find the WB full membrane in Figure S1).

*L. infantum* promastigotes reacted both in vivo and in vitro with the polyclonal antibodies (orb2986) against the conserved 50-amino-acid sequence 350–400 of human TGase 2 inside its catalytic core. Figure 4 shows the indirect immunofluorescent staining of the cells, displaying immunoreactive green spots corresponding to the probable TGase in *Leishmania*. Figure 5 shows the immunoblotting of the extracts from two different strains, revealing one band of ≈74.6 kDa and two bands of ≈66 and ≈54 kDa, respectively (Figure 5, lanes 2 and 3, respectively). Of note, the purified TGase showed reactivity against antibodies in the immunoblotting procedure (Figure 3B). 

## 4. Discussion

While several different isoforms of TGase exist in higher organisms [30,31], the protozoan parasite *Giardia lamblia* possesses three proteins, with both transglutaminase and disulfide isomerase (PDI) activity, of 13, 26, and 50 kDa [32,33]. Regarding *Leishmania*, Soong reported Ca^2+^-independent TGase activity in several *Leishmania* strains [19] and four genome sequences encoding for PDI of 15, 40, 47, and 52 kDa, which can have different subcellular localizations. However, among them, the *L. amazonensis* genes did not show any TGase activity when expressed in *E. coli* [34]. In this study, we report the purification of a 54 kDa TGase from *L. infantum* promastigotes, which showed cross-reactivity with antibodies against a conserved region of the catalytic core of human TGase 2. In addition, we also found bands at 66 and 75 kDa, among which the latter was from a different *L. infantum* strain, suggesting that more than one TGase form can be present based on the considered strain. In fact, while the previous report showed Ca^2+^-independent TGase activity [19], the TGase from *L. infantum* seemed to be modulated by both calcium and GTP. Consistently, GTP at concentrations in the millimolar range inhibits the parasitic *Brugia malayi* TGase with a mass of 56 kDa, which is similar to our purified protein [35]. While a Ca^2+^-independent TGase has been described in *Plasmodium falciparum* parasites [16], other TGases from protists such as *G. lamblia* are Ca^2+^-dependent [32,33]. Thus, we contend that *Leishmania* could have more than one type of TGase, which may not be entirely Ca^2+^-dependent. Unfortunately, since there are still no known genomic sequences of this enzyme, we cannot confirm this hypothesis nor observe whether there has been genomic divergence in the evolution of the enzyme. 

Human TGase 2 is a 77.3 kDa monomer with four domains: an N-terminal β-sandwich domain, a catalytic domain, and two C-terminal β-barrell domains, with a GTP binding site located in a cleft between the catalytic core and the first β-barrell domain [36]. An increased Ca^2+^ concentration reduces the affinity for GTP, causing the exposure of the active site, and Ca^2+^-binding sites are in the core domain [37]. In addition, domains 1 and 2 are the minimum essential structures required for transamidating activity [13]. On the other hand, the absence of domain 4 was shown to cause a loss of GTP binding [13,38]. It is worth noting that the enzyme we found in *Leishmania* is smaller than the human form; thus, it might comprise both Ca^2+^ and GTP domains in a shorter molecule, with possible smaller domains. However, without further experiments on the isolated enzyme (e.g., crystallographic studies), this remains mere speculation. The lack of further characterization of the enzyme in terms of its thermal stability and the pH dependence of its activity may be considered a limitation of this study. Nonetheless, we are confident that despite the differences we found with respect to other reports regarding the modulation of activity by Ca^2+^ and GTP [19], the behaviour might be the same. In fact, Brobey et al. found that the activity of TGase seems dependent on the stage of the parasite, with the shift in the peak of activity from 25 °C in the promastigote to 37 °C in the amastigote [19]. Regarding pH, the majority of TGases, including the one previously found in *Leishmania*, show maximal activity within the range of 8.5 and 9.5 [19,31,35]. Unfortunately, we were unable to test if the enzyme was highly active within the same pH range due to limitations of the used activity assay kit. 

In our opinion, only the protein with an apparent mass of 54 kDa seems to be the “real” TGase in *L. infantum*, which demonstrated transamidating activity after the two-step homogenous purification with chromatography, and it was the most abundant protein form in the preparation, as evidenced by Western blotting experiments. The other two proteins with an apparent mass of 66 kDa and 75 kDa and recognized by TGase 2 antibodies might be proteins with a globular TGase-like catalytic domain. This is typical of the large protein family containing the papain-like peptidase fold, including the *Leishmania* trypanothione synthetase amidase [39], peptide-N-glycanases [40], and proteins important for cell motility [41]. Thus, any of these proteins could have shown positivity induced by the immunostaining procedure. Alternatively, they could be zymogens that require proteolysis to exert activity, as is the case for many TGases [11,18]. 

The metalloprotease GP63, or leishmanolysin, has been identified as substrate of *Leishmania* TGase [19]. GP63 is the most represented protein in exosomes released from the parasite and provides immunosuppressive and pro-parasitic action in the early moments of infection or in the establishment of the associated disease [20,42]. Exosomes were reported for the first time precisely in *Leishmania* [43], and they are also part of the sand fly inoculum [44]. In mammalian cells and *Drosophila*, it has been reported that TGase is secreted via an unconventional Golgi-independent mechanism involving exosomes [45,46]. Furthermore, TGase 2 is associated with both caspase-dependent and caspase-independent cell death [47], and cell death is essential in *Leishmania*, both in the sand fly vector and the mammalian host [48]. For instance, features of programmed cell death such as phosphatidylserine exposure at the cell surface are used by the parasite to enter neutrophils or macrophages, which recognize their target and engulf it [49]. At the same time, this causes the phagocyte to produce immunosuppressive cytokines, allowing the parasite to survive and grow [50]. Thus, TGase might be involved in parasites’ infectivity and may represent a new virulence factor, which is possibly present in *Leishmania* exosomes with GP63. This hypothesis will be addressed in the future by employing more sophisticated techniques, such as 2D electrophoresis and mass spectrometry, that will help identify a sequence useful for cloning the enzyme. As a further step, the physiological functions of this TGase should be explored in order to determine its potential differences from mammalian TGase and open new avenues for it use as a possible drug target. Moreover, testing its antigenicity might unveil whether it can be useful in diagnosis or vaccine studies.

In conclusion, we have shown that *L. infantum* possesses a Ca^2+^- and GTP-dependent TGase with an apparent mass of 54 kDa, which can be purified by cation exchange and affinity chromatography on immobilized heparin. By employing polyclonal antibodies against a 50-amino-acid conserved region of the catalytic core of human TGase 2, we also revealed two other bands of a higher mass that might be TGase-related proteins or other variants of the enzyme. 

## Data Availability

The data presented in this study are available on request from the corresponding author.

## Supplementary Materials

The following supporting information can be downloaded at: https://www.mdpi.com/article/10.3390/vetsci10030234/s1; Figure S1: WB full membrane for Figure 3; Figure S2: WB full membrane for Figure 5.
