# Supplementary figures and images for: A Calcium- and GTP-Dependent Transglutaminase in Leishmania infantum
Source: Vet Sci. 2023 Mar 20;10(3):234. doi: 10.3390/vetsci10030234 (PMC10053793; doi:10.3390/vetsci10030234)

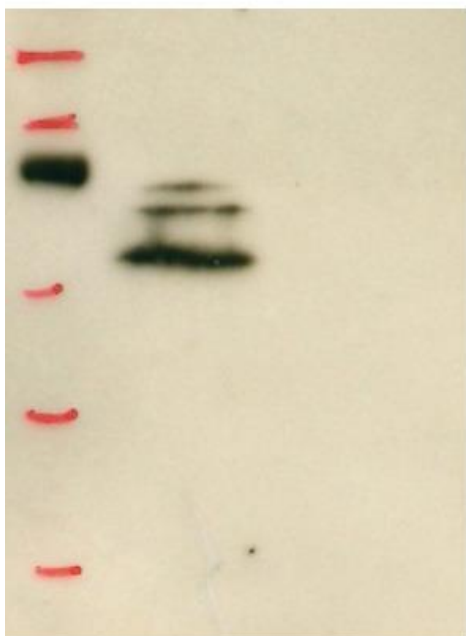

**Figure S1.** WB full membrane for Figures 3.

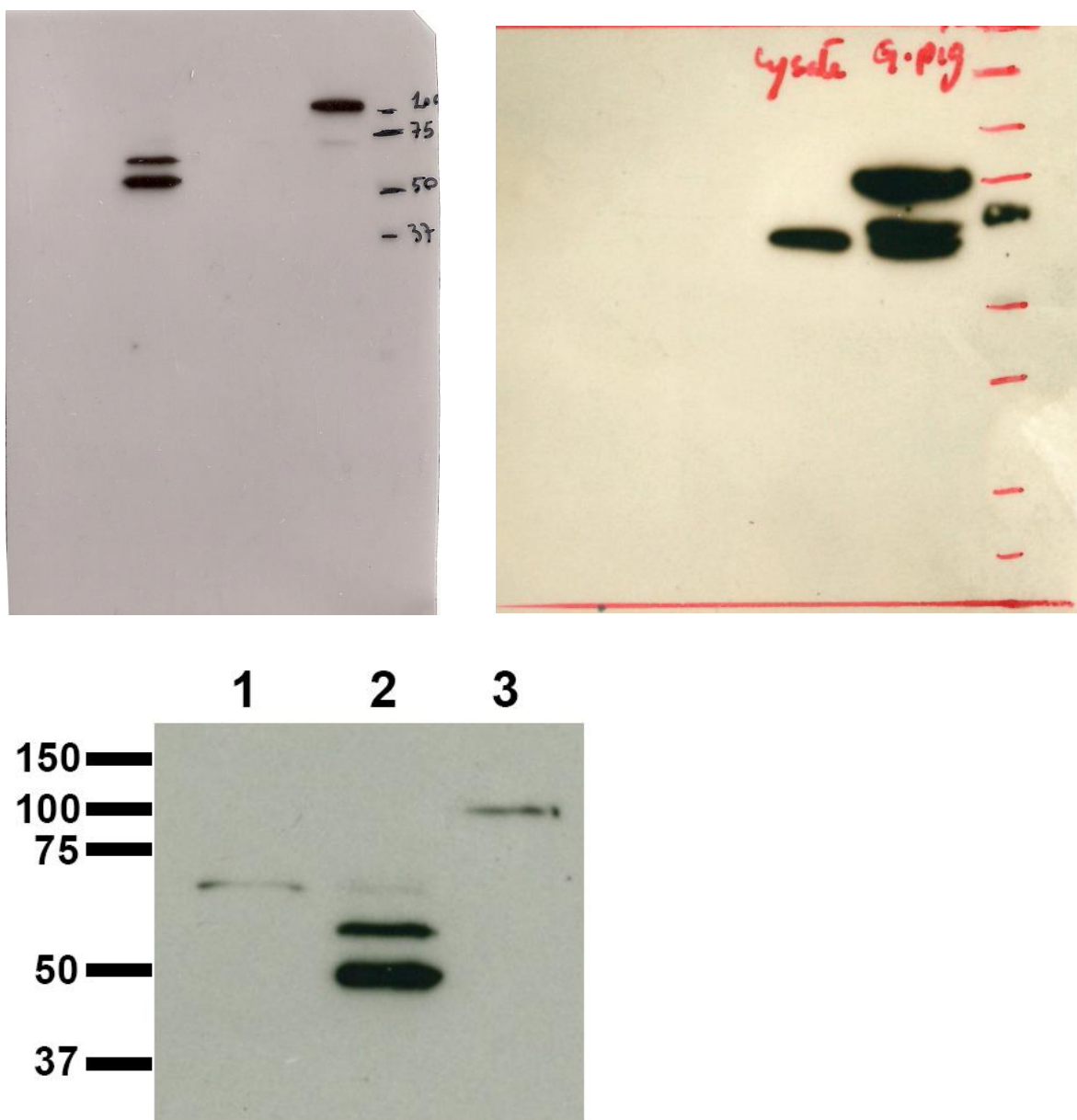

Figure S2. WB full membrane for Figures 5.

Supplement: Supplementary file 1 [file vetsci-10-00234-s001.zip › vetsci-2269348-SM.pdf]
